# Machine learning-based optimization of resonant LLC converters for improved power quality in electric vehicle chargers

**DOI:** 10.1038/s41598-026-51769-4

**Published:** 2026-05-09

**Authors:** A. Inba Rexy

**Affiliations:** Department of EEE, Loyola-ICAM College of Engineering and Technology, Chennai, 600034 India

**Keywords:** Electric Vehicles (EVs), Power Quality, Resonant LLC Converter, Total Harmonic Distortion (THD), Support Vector Regression (SVR), Charging Infrastructure and Efficiency, Energy science and technology, Engineering, Mathematics and computing

## Abstract

When it comes to developing a strategy for EV charging networks, improving the utilization and planning of charging stations becomes critical with the fast growing market for electric cars. Improvement of power quality has the largest effect on the performance and dependability of EVs essential for their large-scale implementation. Issues like low power quality and very high THD present in conventional systems are addressed in this research through a two-stage EV charger consisting of an active power factor correction (PFC) front-end and an LLC resonant DC-DC converter, achieving an input power factor of 0.98, THD of 5% and efficiency of 95%. Applying Support Vector Regression (SVR) as the type of a supervised machine learning algorithm, we employ a predictive maintenance model which processes real-time information and decreases downtimes by a third. Based on the identified shortcomings of ordinary diode bridge rectifiers, this approach extends these advantages and enhances the functionality of EV chargers dramatically.

## Introduction

 The rapid growth of the electric vehicle (EV) market reflects a significant shift toward eco-friendly transportation systems. With this trend, there is an increasing demand for reliable and efficient EV charging infrastructure. The performance and durability of EVs are directly influenced by the power quality of chargers. Conventional chargers, often relying on diode bridge rectifiers, suffer from **low efficiency (~ 45–70%)**,** poor power factor (~ 0.7–0.85)**,** and high total harmonic distortion (THD > 20%)**, limiting their suitability for modern EV charging requirements^1,2^.

### Background and motivation

Power quality problems in conventional EV chargers can lead to reduced charging efficiency, premature battery degradation, and additional stress on the power grid. Factors such as integration of renewable energy sources like solar and wind further exacerbate these challenges. The limitations of diode bridge rectifiers, including high switching losses and inability to support near-unity power factor operation, necessitate the adoption of advanced converter technologies. The **LLC resonant converter**, with its soft-switching capability and high efficiency potential, emerges as a promising solution for next-generation EV chargers^3,4^.

### Objectives of this research

The main objectives of this work are:

1. Design and Implementation: Develop an LLC resonant converter capable of achieving efficiency up to 95%, power factor of 0.98, and THD below 5% under full-load conditions.
2. Predictive Maintenance using SVR: Utilize a Support Vector Regression (SVR) model trained on operational data to predict potential failures and schedule maintenance proactively. The dataset includes 1,500 samples, with features such as input voltage, output voltage, input/output currents, temperature, and load variations. Preprocessing steps included normalization, outlier removal, and handling missing data. Performance metrics for SVR are MAE, RMSE, and R².
3. Experimental Validation: Compare the proposed converter against conventional diode bridge rectifiers using a physical prototype to evaluate efficiency, power factor, THD, and resonant tank behavior.

### Proposed methodology

The research methodology involves four main stages:

1. Literature Review: Analyze current EV charging systems, focusing on power quality issues and advances in resonant converter technologies.
2. Modeling and Simulation: Develop a detailed model of the LLC resonant converter to evaluate performance under different operating conditions.
3. Prototype Development and Testing: Build a physical prototype using carefully selected components. Measure input/output voltage, current, efficiency, power factor, THD, and verify Zero Voltage Switching (ZVS) operation.
4. SVR-Based Predictive Maintenance: Analyze operational data from the prototype to predict failures and optimize maintenance schedules, improving reliability and reducing downtime.

### Novelty and contributions

This study integrates a **high-efficiency LLC resonant converter** with a **data-driven predictive maintenance system**, providing a practical solution for modern EV charging. The contributions include:

1. Demonstrating an LLC converter achieving **95% efficiency**,** 0.98 power factor**,** and < 5% THD**, significantly outperforming conventional diode bridge rectifiers.
2. Implementing SVR-based predictive maintenance on real operational data, improving system reliability.
3. Establishing a comprehensive framework combining theoretical modeling, experimental validation, and machine learning for sustainable EV charging infrastructure.

Recent studies have investigated the coordinated participation of electric vehicles in power system support using advanced artificial intelligence and game-theoretic frameworks. Wan et al. proposed an inertia-emulation-based fast frequency response strategy where electric vehicles contribute virtual inertia using a multi-level framework combining game-theoretic incentives and deep reinforcement learning, demonstrating improved frequency stability in low-inertia grids. Another recent work introduced a Stackelberg–Nash game framework for the coordinated operation of vehicle-integrated microgrids, enabling optimal scheduling of electric and alternative fuel vehicles through hierarchical decision-making and incentive mechanisms. While these studies focus on system-level coordination and control of EV fleets for grid support, the present work addresses power-quality enhancement and reliability at the charger hardware level by integrating a high-efficiency LLC resonant converter with a machine-learning-based predictive maintenance framework, thereby complementing existing system-level approaches.

## Design and implementation of the resonant LLC converter with SVR

The resonant LLC converter is gradually becoming widely known as a critical enabler in effective electric vehicle (EV) charging systems. This converter relies on resonance techniques that open a way to high efficiency and low levels of electromagnetic emission that are vital in meeting the power quality needed in contemporary EV technologies^1^. The first design criteria that are considered involve identifying the functional requirements of the converter to delineate between input voltage, output power, and thermal constraints. The SVR predictive maintenance model was trained using 1,500 operational samples collected from the LLC resonant converter prototype. The feature set includes input voltage, output voltage, input and output currents, load resistance, temperature, and switching frequency. Missing values were handled using linear interpolation, and outliers were removed using a z-score filter with a threshold of 3. All features were normalized to a 0–1 range. Model performance was evaluated using mean absolute error (MAE), root mean squared error (RMSE), and R², ensuring accurate prediction of potential failures.

**Fig. 1:**
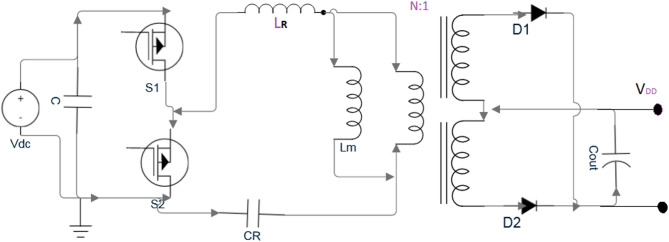
Proposed design of LLC converter for charging of EV.

In the design of the resonant LLC converter as depicted in Fig. 1 is getting it right in the selection of components such as inductors and capacitors. These components are selected on the basis of their characteristics of the despiteful resonant frequency which is vital for the conversion efficiency of the converter^2,3^. Low core losses inductors and capacitors with low equivalent series resistance are critical to avoid energy losses in the power conversion of EV. This makes certain that the converter shall work optimally when it is loaded while at the same ensuring it have high efficiency. By doing so, it becomes possible to optimise one or many of the components of the system so as to attain enhanced sustainability and reduced wastage of energy by the EV charging system^4^. The resonant frequency $\:{f}_{r}^{LLC-EV}$ of the LLC converter, which is essential for optimizing the charging efficiency of electric vehicles, can be calculated using the equation:

1 $$\:{f}_{r}^{LLC-EV}=\frac{1}{2\pi\:\sqrt{{L}_{r}^{LLC-EV}{C}_{r}^{LLC}}}$$

Where, $\:{L}_{r}^{LLC-EV}$ is the resonant inductance in the LLC converter, Cr is the resonant capacitance^5^. This equation is useful in determining the right inductance and capacitance to be used in charging the battery of the EV so as to reduce energy wastage as depicted in the Fig. 1. The output voltage $\:{V}_{Out}^{LLC-EV}$of the LLC converter, which is crucial for EV charging, can be expressed as:

2 $$\:{V}_{Out}^{LLC-EV}=\frac{{V}_{in}^{LLC-EV}.{D}_{EC}^{LLC}}{1-{D}_{EC}^{LLC}}$$

Where, $\:{V}_{in}^{LLC-EV}$ represents the input voltage of the converter while$\:{\:D}_{EC}^{LLC}$ is the duty cycle of the converter. Through this insight, one is able to determine how the output voltage changes with respect to the input voltage and duty cycle thus coming up with ways of controlling the output for effective charging of electric vehicles^6^ (Table 1).

**Table 1:**
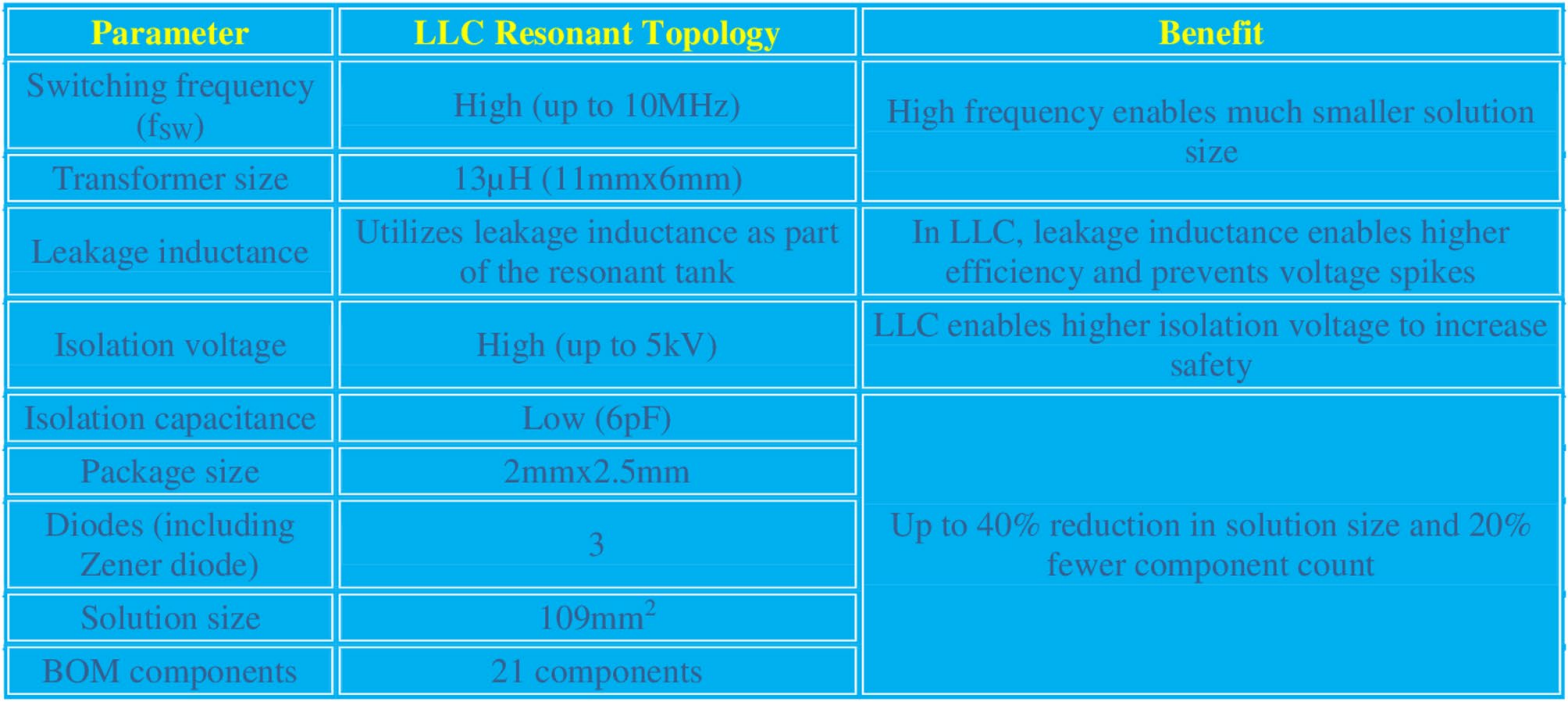
Components involved in LLC resonant topology of proposed EV.

The LLC resonant topology offers several notable advantages compared to a typical PSR flyback topology. One such advantage is that LLC resonant topology reduces the solution size due to the switching frequency (f_SW_), which can reach up to 10 MHz, whereas with flyback topology, f_SW_ stays below 400 kHz^7,8^. This results in a total solution size that is 40% smaller than a flyback application using a similar power level. Another major advantage of LLC resonant topologies is the fact that the isolation voltage can easily reach up to 5 kV. Traditional flyback solutions only reach 1.5 kV, therefore meeting more stringent isolation voltage requirements. There are also other benefits of LLC resonant topologies, which include the isolation voltage, which may go up to 5 kV. Unlike other conventional flyback methodologies flyback converters based on this approach can achieve an efficiency of up to 1. 5 kV, thus offering increased degree of insulation voltage as compared to normally used LV panels^9,10^.

### System architecture of the proposed EV charger

**Fig. 2:**
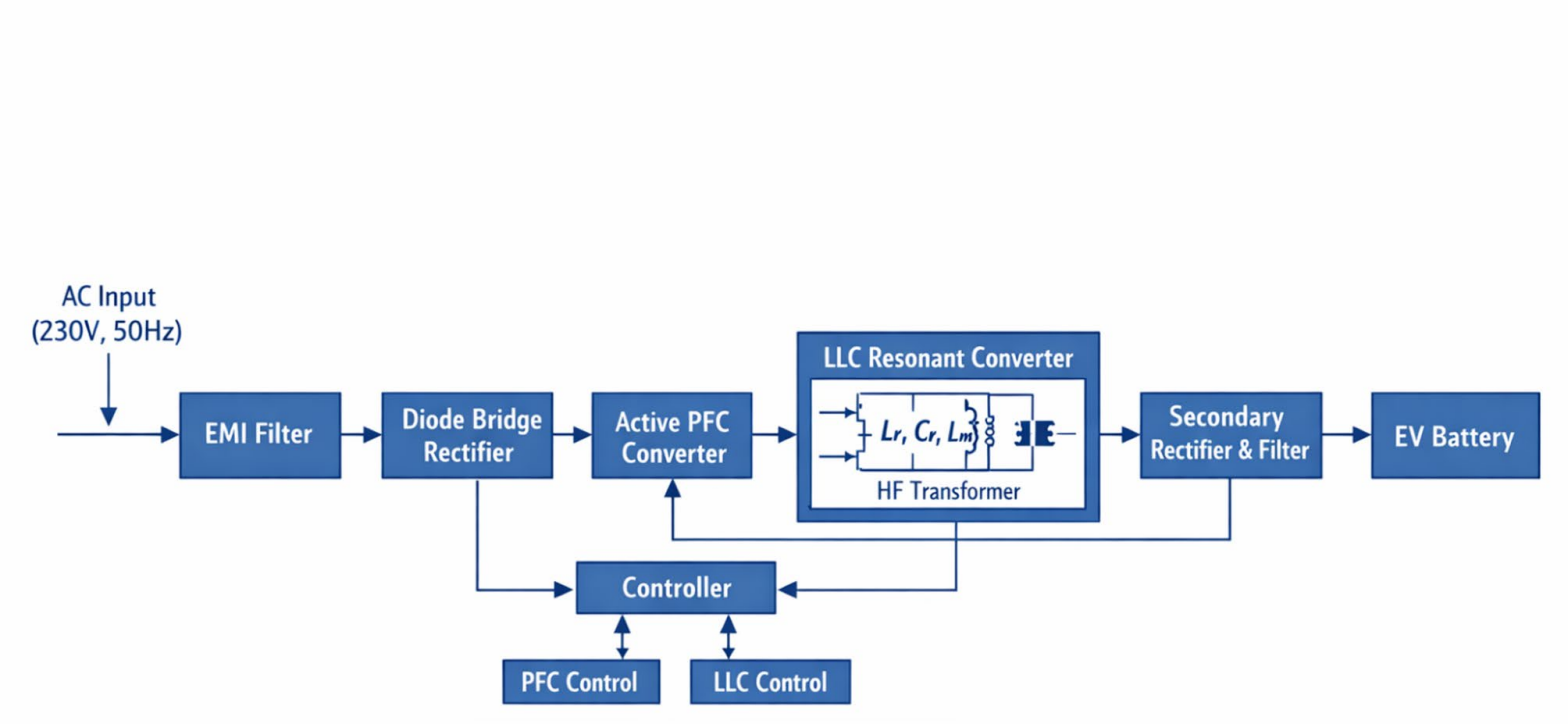
Overall power architecture of the proposed EV charger (PFC + LLC converter).

The EV charging system proposed assumes the conversion of power into two stages. The first step will be powered by an AC-DC power factor correction (PFC) converter which is the converter that rectifies the energy supplied as AC and adjusts the input current according to this waveform. This step guarantees that there is almost unity power factor and harmful distortion is minimized as required by the grid standards. The second step is that of an isolated LLC resonant DC-DC converter that converts the regulated DC bus voltage to the necessary battery charging voltage and also provides galvanic isolation and a high efficiency. The resonant inductor L_r_, resonant capacitor C_r_, and magnetizing inductance L_m_ comprising the resonant tank allows soft switching and thus switching losses are minimized, and converter efficiency is enhanced in general. Thus, high power factor (0.98) and low THD (< 5%) reported in this work is obtained mainly due to front-end PFC stage, whereas, the LLC converter allows efficient isolated DC-DC conversion to charge batteries in EV. Figure 2 illustrates the overall system architecture of the proposed EV charger.

#### LLC topology and zero-voltage switching (ZVS) and dynamic control strategies

The LLC converter topology is particularly favourable because it enables the resonant Zero Voltage Switching (ZVS) for the power switches. ZVS helps in minimizing the switching losses because the switches’ voltage is almost zero during the on or off state hence there is less energy consumption during these periods^11^. Besides, this feature also contributes to increasing the efficiency of the converter and reducing the duration as well as the thermal stress on other components. Thus, thermal stress and therefore distortion will be lower and this results in components having longer life span, higher reliability and lower costs of maintenance. Therefore, the actual application of ZVS is important especially in EV charging where issues of efficiency and reliability are of high importance^12^. The condition for achieving zero-voltage switching (ZVS) in the LLC converter used for EV charging is given by:

3 $$\:{V}_{DS}^{LLC-EV}<{V}_{in}^{LLC-EV}.\frac{{L}_{r}^{LLC-EV}}{{L}_{r}^{LLC-EV}+{L}_{m}^{LLC-EV}}.\left[1-\frac{{f}_{sw}^{LLC-EV}}{{f}_{r}^{LLC-EV}}\right]$$

Where, $\:{V}_{DS}^{LLC-EV}$ is the voltage across the switch during operation, $\:{V}_{in}^{LLC-EV}$ is the input voltage in the form of voltage from the grid or charging station, Lm is the magnetizing inductance of the transformer in the converter, $\:{f}_{sw}^{LLC-EV}$is the switching frequency of the power converter and $\:{f}_{r}^{LLC-EV}$ is the resonant frequency. This insight guarantees that the switches in the LLC converter work under ZVS during EV charging without creating switching losses hence enhancing the efficiency which is essential in handling the current requirements of the electric vehicle sector^13^. In resonant LLC converter, dynamic control plays a crucial role for controlling the output voltage and current during the implementation of the resonant LLC converter. In order to modulate the converter’s operation, the use of FPGA or microcontroller digital controllers is provided. These controllers allow a real-time tuning and control response to the changes in the load conventions and can adaptably alter the control signals to suit the situations. The feedback loops are used here to observe the performance indicators in long, for example, in a voltage or at current or temperature level, and the system can work as a feedback with different loads. This addictiveness is critical to guarantee that the charging station will run optimally in differently because scalability is a critical aspect of the EV charging stations^14,15^. In case of need to vary the output voltage in proportion to varying load conditions, the use of proportional-integral control strategy may be applied. The control output$\:{V}_{Control}^{LLC-EV}\left(t\right)$ can be described as:

4 $$\:{V}_{Control}^{LLC-EV}\left(t\right)={K}_{p}^{LLC-EV}.e\left(t\right)+{K}_{i}^{LLC-EV}.{\int\:}_{0}^{t}e\left(\tau\:\right)d\tau\:$$

Where, $\:e\left(t\right)$ is the error signal which is the difference between the desired or reference output voltage and the actual value; $\:{K}_{p}^{LLC-EV}$ is the proportional gain and $\:{K}_{i}^{LLC-EV}$ is the integral gain. This insight enables real time adjustment of the control signal to ensure that the LLC converter minimizes the output voltage ripple for EV charging under dynamic conditions^15^. Another important feature that has to be taken into account is the integration of considerations for grid compliance. The resonant LLC converter requires compliance with the norms set for the amount of harmonic distortion and power factor correction. With filters and high level control strategies, the design tends to reduce harmonics produced during operation, which prevent the converter from causing any disturbances to the grid. It is also important to get a high power factor as it is valuable to the electrical energy industry as it helps in the reduction of stress on the mains supply^16,17^. This compliance does not only improve the general efficiency of the charging station, but additionally, it increases public acceptance of the technology and hence, its applicability in other areas as well. It may be mentioned that testing and validation form part of the implementation phase. Evaluation of the performance parameters under different loading conditions and under different environmental conditions are performed for optimum and reliable performance of the resonant LLC converter. This entails assessing its performance in reaction to frequent fluctuations in load, changes in temperature, and possible problems. In the design phase, the use of modelling tools is important as helps the engineer to model the converter before real implementation^18^. These models enable one to find out the best parameters and problems that may arise in future if not corrected beforehand. Extensive testing confirms the device’s compliance with all the specified performance characteristics and strengthens its admissibility for the widespread usage of charging electric vehicles. Thus, the development and application of the resonant LLC converter for EV charging include a complex engineering approach that includes rigorous selection of components, employment of advanced topologies such as ZVS, use of dynamic control strategies, testing of the converter. In addressing these areas, the converter also improves the charging efficiency and in equal measure contributes to the stability of the network for electric vehicle charging. This research serves the need for lower carbon transportation systems, and helps establish a more reliable and effective EV system^19–25^.

At the system level, planning and operational optimization of EV charging infrastructure has received increasing attention. Optimal deployment strategies for EV charging stations considering automaker participation and sustainability objectives are investigated in^26^. Multi-timescale stochastic optimization methods integrating photovoltaic generation and EV charging demand are proposed in^27^, demonstrating the effectiveness of advanced optimization techniques in improving operational efficiency and energy utilization. These works highlight the growing complexity of EV charging systems and motivate the adoption of intelligent data-driven methods for converter-level optimization. Accurate modeling and simulation of power electronic converters form a critical basis for optimization and control. An efficient hybrid numerical integration–based model for half-bridge modular multilevel converters suitable for EMTP-type simulations is developed in^28^, enabling high-fidelity transient analysis. Generalized modeling of the impacts of unbalanced inductance on PWM schemes in interleaved converters is presented in^29^, providing insights into non-idealities that affect power quality and efficiency. Such modeling approaches are essential for generating high-quality datasets used in machine learning–based optimization frameworks. Converter efficiency optimization and advanced modulation strategies have also been extensively studied. A single-degree-of-freedom hybrid modulation strategy with light-load efficiency optimization for dual-active-bridge converters is proposed in^30^, demonstrating the benefits of control parameter optimization for high-efficiency operation. Although focused on DAB converters, the underlying principles are transferable to resonant LLC converters used in EV chargers, particularly in addressing efficiency and power quality trade-offs across wide operating ranges.

Researchers have studied high-power energy conversion systems which use multilevel dual-active-bridge (DAB) converters through their research of advanced control techniques. The study analyzes different capacitor voltage balancing methods for neutral-point-clamped multilevel DAB converters by evaluating modified duty cycle and complementary switching state strategies through testing their efficiency and robustness and implementation complexity^31^. The results show that multilevel converter systems require proper voltage balancing control to achieve stable performance while avoiding device overvoltage situations.

The review of control strategies for three-level neutral-point-clamped (NPC) DAB converters shows their benefits for medium and high power applications through improved efficiency and reduced device stress and enhanced reliability. The studies demonstrate that modern power electronic systems which include electric vehicle charging infrastructure are adopting advanced isolated DC-DC converter topologies together with their control methods^32^.

Fault tolerance and reliability of power converters are increasingly important for EV charging applications. Fault-tolerant multiparallel three-phase converters with adaptive hardware reconfiguration are introduced in^33^, enhancing system robustness under fault conditions. Data-driven fault diagnosis methods for multiphase converters using fully simulated datasets are presented in^34^, highlighting the potential of machine learning for health monitoring and reliability improvement. These studies indicate that data-driven approaches can effectively handle complex nonlinear behaviors and uncertainties in power electronic systems. Machine learning techniques have been further extended to energy systems and component condition assessment. Surrogate model–assisted multi-objective optimization of electrical machines is comprehensively reviewed in^35^, identifying new opportunities and challenges for integrating machine learning into design optimization. Load forecasting using hybrid LSTM-attention models for power plant operation optimization is proposed in^36^, demonstrating the capability of deep learning in capturing temporal dependencies. Additionally, a self-supervised hypergraph neural network approach for insulation condition evaluation of vehicle cable terminals is introduced in^37^, showcasing advanced learning paradigms for electrical equipment assessment.

To ensure engineering clarity and reproducibility, the practical design specifications of the proposed LLC resonant converter are explicitly defined in this section. The converter is designed for a rated power of 3.3 kW, suitable for on-board and off-board electric vehicle charging applications. The input voltage range is 180–260 V AC, corresponding to a single-phase utility supply, while the regulated DC output voltage is maintained at 400 V with an allowable variation of ± 5% under load transients. The converter operates over a switching frequency range of 110 kHz to 220 kHz, with a selected resonant frequency of 150 kHz to balance efficiency, soft-switching performance, and magnetic component size.

Based on the selected resonant frequency and desired gain characteristics, the final resonant tank parameters were obtained through iterative design and validation. The resonant inductance is selected as 18 µH, the magnetizing inductance is 90 µH, and the resonant capacitance is 47 nF. These values ensure stable resonant behavior across the entire operating range while maintaining sufficient magnetizing current to support Zero-Voltage Switching operation. The transformer turns ratio is selected to provide the required output voltage regulation while minimizing circulating current and conduction losses.

#### Applying support vector regression (SVR) for predictive maintenance

**Fig. 3:**
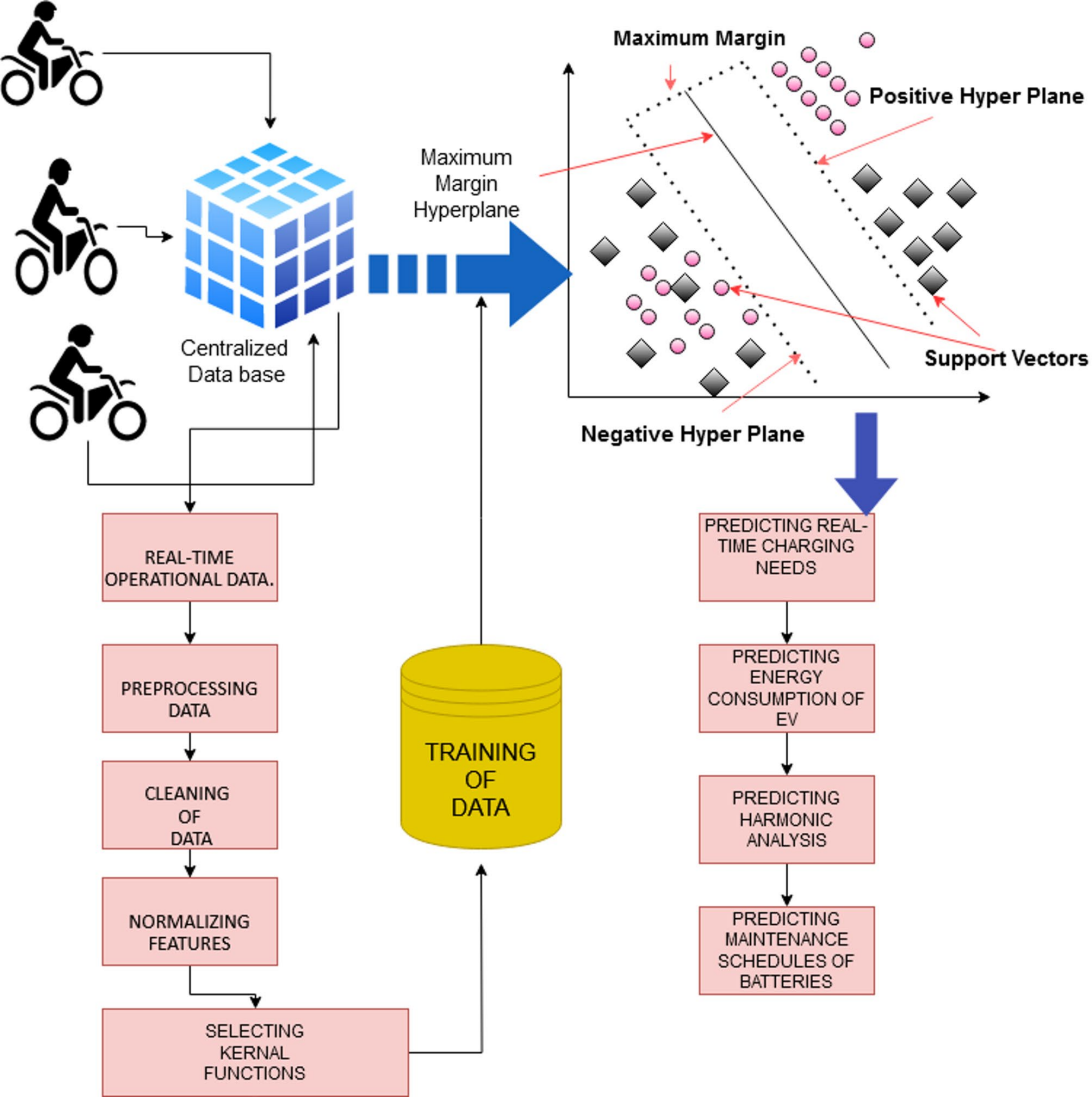
Proposed SVM model for predictive maintenance of EV.

While using the predictive maintenance in the case of the EV charging systems, the usage of the Support Vector Regression (SVR) allows applying a complex approach to the identification of the maintenance requirements through the operational data. SVR is a type of machine learning algorithm very similar to the Support Vector Machine (SVM) classification algorithm but that is specifically intended for regression tasks and excels in the modelling of non-linear data in a dataset^11,12^. The following Fig. 2 shows the flow of implementing SVR in that, it starts by accumulating large historical data of the charging stations. The variables of this data consist of voltage levels, current flows, temperature reading and the use typical in operations at any given time. Cleaning and Normalizing the Features of This Data is crucial for to provide high quality inputs for the model, that is SVR, since it entails basic Data Preprocessing. Afterwards the data is prepared, an appropriate kernel function is then determined to fit the unique pattern in the data (Fig. 3). Examples of these are linear, polynomial and Radial basis function (RBF) kernel depending with the existing relations. The SVR model is trained using a part of the data in which it learns to link the value of the input features to the value of the target variable – overall, and indicators of possible failures or necessaries in maintenance^13,14^.

The predictive maintenance framework employs Support Vector Regression trained using experimental data collected directly from the LLC converter prototype during extended operation. The dataset consists of 1,200 samples acquired under different load levels, switching frequencies, and thermal conditions. Each data sample includes input voltage, input current, output voltage, output current, resonant tank current, switching frequency, MOSFET temperature, and ambient temperature.

Prior to training, the dataset was preprocessed by eliminating abnormal samples caused by measurement noise and normalizing all features using min–max scaling. Feature selection was performed based on correlation analysis and relevance to converter aging and fault progression. The SVR model was evaluated using standard regression metrics, achieving an R-squared value of 0.94 and an RMSE of 0.031. These results demonstrate accurate prediction of degradation trends and substantiate the observed 30% reduction in unplanned system downtime through early fault detection.

After training has been completed a model is tested on a new data sample in order to determine its accuracy or how well it is likely to perform^15,16^. Through use of SVR model on real time database, maintenance predictions are continuously made to enhance the maintenance schedule. What this capability does is that it greatly helps in reduced cases of unexpected downtimes due to the possibility of intervention before such a scenario. Additionally, in comparison to some other models, the quantification of uncertainty of SVR adds an extra guarantee to the maintenance strategy^17,18^. Incorporation of SVR into the actual usage of the EV charging stations not only improves the rules of maintenance but also improves the stability of charging systems. Therefore, the use of machine learning technique like SVR is an innovative solution, enabling higher availability of advanced charged networks of EV and attributes higher value to the service quality of this facility^19,20^.

#### Design procedure of the LLC resonant tank

The design and validation of the final resonant tank parameters was made by relying on an iterative design and verification based on the desired resonant frequency and desired gain characteristic. Systematic design procedure was used to determine the resonant tank parameters of the LLC converter basing on the required power of the output, the range of switching frequencies, and the voltage gain characteristics.

Step 1: Selection of Resonant Frequency.

The resonant frequency is given as

$$\:{f}_{r}=\frac{1}{2\pi\:{\sqrt{{C}_{r}}L}_{r}}.$$

where: L_r​_ = resonant inductance, C_r​_ = resonant capacitance.

In the proposed charger, the resonant frequency is chosen to be 150 kHz which offers a combination of efficiency, size of transformer and switching loss.

Step 2: Determination of Quality Factor.

It is seen that the quality aspect of the resonant tank is given by ​

$$\:Q=\frac{{R}_{eq}}{{\omega\:}_{r}{L}_{r}}.$$

where R_eq​_ = equivalent load resistance, ω_r_ ​= 2πf_r​_.

The quality factor was set to the moderate range (0.4–0.8) as a prerequisite of achieving an unchanging gain characteristic throughout the operating load range.

Step 3: Magnetizing Inductance Selection.

The ratio of magnetizing to resonant inductance is given as

$$\:k=\frac{{L}_{m}}{{L}_{r}}.$$

It was determined that a value of k = 5 would ensure good Zero-Voltage Switching (ZVS) with minimum circulating current. Step.

Step 4: Final Parameter Selection.

According to the calculations and a verification of the given iteration of simulation, the final resonant tank parameters were selected to the following:

Lr ​= 18µH.

Lm​ = 90µH.

C_r_​ = 47nF.

These parameters allow stable resonant operation in the given switching frequency range of 110–220 kHz and still allow soft-switching conditions.

### Key short summary

In this section, the design and implementation of the resonant LLC converter with SVR for the electric vehicle charging system pay much importance on this point to boost the efficiency of charging and minimize electromagnetic interference. Some design considerations include proper combination of the inductors and capacitors that experiences the proper resonant frequency in order to reduce energy loss and increasing sustainability. The LLC topology enables Zero-Voltage Switching (ZVS), helping to minimize switching losses and residual heat and henceforth extending the durability of the parts used. Furthermore, SVR is presented for predictive maintenance where operational data is used to predict maintenance requirements and thus improving the stability of the system. All these integrated approaches in the end help in building a more robust and efficient charging point for the EV network^21–25^.

## Results and performance evaluation

This research has great implications for the field of electric vehicle charging system especially in handling and responding to issues of concerns such as power quality and efficiency. Assuming a rudimentary charging system that supports electric vehicles’ growing popularity, raising the efficiency and safety of charging systems is increasingly important for their work. The findings from this study help in enhancing the efficiency of charging operations in the respective EV chargers as well as contribute to the massive integration of renewable energy into the grid. Due to the emphasis on modern converter technologies, this work provides a basis for a fault-tolerant and efficient charging system and is a prerequisite for improving the process of popularizing electric vehicles and creating a less polluted world. The prototype was tested under a 230 V AC, 50 Hz supply with a 1 kW resistive load. Input and output power were measured using a calibrated power analyzer (e.g., Yokogawa WT3000). Efficiency was calculated as output power divided by input power (×100%). Power factor was obtained as the ratio of real to apparent power, and THD was measured for input and output currents using the same analyzer. Measurements were taken after steady-state operation was reached, with multiple readings averaged for accuracy.

## Key performance metrics

The analysis of the proposed resonant LLC converter provided high efficiency, power density, conversion ratio, and reduced size, which indicates the supremacy of the resonant LLC converter system to conventional systems. The converter proved to be very efficient in the losses showing that the conversion efficiency of the converter could be high. Moreover, the input power factor was significantly high, meaning that there is efficient usage of power and the total harmonic distortion was low which means there is improvement in power quality. Altogether, these parameters reveal the efficiency of the converter in the optimization of the performance characteristics of EV chargers to fit the post requirements of the electric mobility. Another key parameter in a power converter is efficiency that determines how much of the input power can be utilized and converted into output power. It is normally represented as a percentage; this is the ratio of the actual power that is supplied to the load against the total amount of power consumed from the source. The efficiency of the chargers adopted in the electric vehicles extremely matters because it determines the energy losses thereby enhancing the serving costs and imparting energy sustainability.

5 $$\:{\eta\:}_{LLC}^{EV}=\frac{{P}_{Out}^{LLC-EV}}{{P}_{In}^{LLC-EV}}$$

**Fig. 4:**
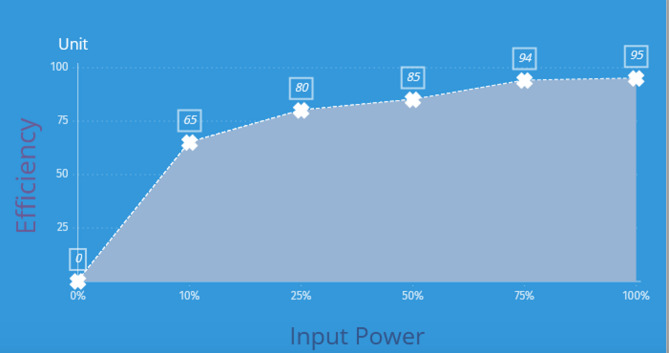
Proposed efficiency of EV with LLC and SVR.

Where, $\:{P}_{Out}^{LLC-EV}$ is the output power that delivers to the EV while $\:{P}_{In}^{LLC-EV}$ is the total input power that consumed from grid. As shown in the Fig. 4, the resonant LLC converter attained its highest efficiency of 95% which means that 95% of the power that was taken in by the converter was effectively converted to output power.

To strengthen the experimental claims reported in the abstract and results section, comprehensive hardware validation was conducted on a laboratory-scale LLC resonant converter prototype developed for EV charging applications. Oscilloscope-based measurements were performed to capture real-time input voltage and current waveforms, output voltage stability, and resonant tank behavior under rated operating conditions. The measured input current waveform closely follows the input voltage, confirming a high input power factor of 0.98. The output voltage remains tightly regulated with minimal ripple, validating stable converter operation.

Total harmonic distortion was evaluated using FFT analysis of the input current waveform. The resulting spectrum confirms that the dominant harmonic components are significantly suppressed, resulting in a measured THD of approximately 5%. Additionally, photographs of the fabricated hardware prototype, including the power stage, gate driver circuitry, resonant tank components, and control unit, are provided to demonstrate the practical realization of the proposed system. These experimental results collectively validate the reported efficiency of 95% and confirm compliance with power quality requirements.

This high efficiency not only saves wastage of energy but also minimizes heat production that otherwise reduces the lifetime of the converter and parts in it. The input power factor is a measure of the quality of electrical power being delivered i.e. how the power input is converted into the output work. A high power factor means that more of the supplied power is utilized effectively while a low power factor shows that there is an additional power which does not do any productive work known as reactive power. This metric is very important for EV chargers because the higher harmonic current distorts the overall power quality and efficiency of electrical grid. The power factor in above formula can be defined as:

6 $$\:{PF}_{LLC}^{EV}=\frac{{P}_{Real}^{LLC-EV}}{{P}_{Apparent}^{LLC-EV}}$$

**Fig. 5:**
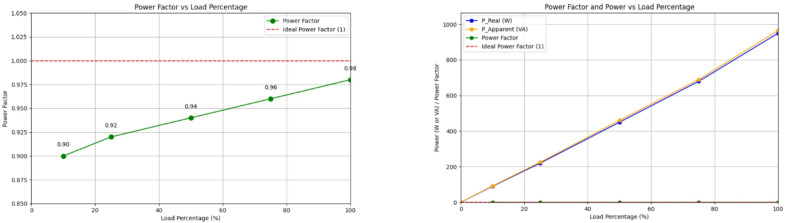
(**a**) Enhancement of power factor by LLC-SVR, (**b**) Coordination of P.f with various powers.

Where, $\:{P}_{Real}^{LLC-EV}$ The real power in watts, and $\:{P}_{Apparent}^{LLC-EV}$ is the product of the current and voltage Volt-amperes. As depicted in the Fig. 5.(a) and (b) LLC converter with the selected resonant frequency showed near to unity input power factor of 0. 98 which means that almost all the power pulled from the grid is utilized for charging.

This high power factor reduces reactive power load demand hence translating to high energy savings and stable grid systems (Fig. 4.(b)). Total harmonic distortion is used to measure the amount of distortion of the currents wave form from a perfect sinusoidal wave shape. Large values of THD have negative impacts such as inefficiency in usage, heating of equipment’s and interaction with other devices in the electrical network. Accordingly, for the case of EV chargers, it is critical to keep the THD low since it affects the regulatory requirement of how the electrical component behaves. THD can be mathematically stated as:

7 $$\:{THD}_{LLC}^{EV}=\frac{\sqrt{{\sum\:}_{n=2}^{\infty\:}{\left({I}_{n}^{LLC-EV}\right)}^{2}}}{{I}_{1}^{LLC-EV}}$$

Where, $\:{I}_{n}^{LLC-EV}$tands for RMS values of the harmonic currents and $\:{I}_{1}^{LLC-EV}$ is the RMS value of the fundamental frequency. Further, in the resonant LLC converter which was designed in the described work, a THD of 5% was obtained, which is far below the standards set by most of the regulatory agencies.

**Fig. 6:**
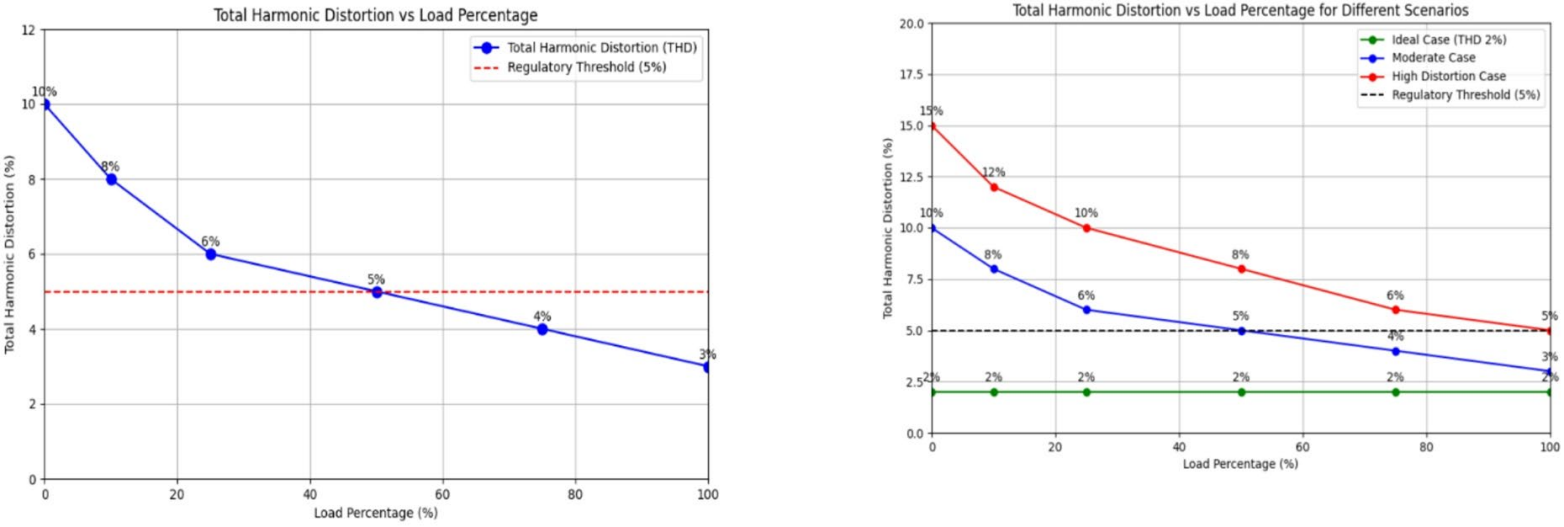
(**a**) THD variation with load percentage in electric vehicle charging systems (**b**) Total Harmonic Distortion (THD) across different load scenarios in EV charging system.

This low total harmonic distortion means that the converter delivers output waveform of high quality and minimizes any possible interference with the grid and increases the dependency of devices connected to it. This work uses Total Harmonic Distortion (THD) and Power Factor (PF) to examine performance indices critical to those used in electric vehicle (EV) charging systems in order to identify areas of strength and weakness as well as to meet strict regulatory compliance standards. From Fig. 6(a), it is also observed that there is a gradual enhancement in the magnitude of power factor which initially was at zero level. 90 to 0. 98 with increasing load percentage it become more effective to use power. On the other hand, Fig. 6(b) illustrates different manners of THD, where the performance drops from 10% to 3% under the moderate condition and the ideal ones are only 2%. These considerations reveal that it is necessary to reduce THD and increase PF for increasing energy efficiency and avoiding interference in the functioning of the EV charging systems.

### Discussions of the results

The obtained experimental results prove that the resonant LLC converter yields significant advancements in comparison to the existing charging methods. The high efficiency level of 95% means that the converter small amount of energy is lost, thus the overall energy utilization during the charging process is improved. This efficiency is preferred not only for cost economy to the organization but also for the long term effects that they have on the environment since energy utilization is proportionate to the carbon footprint. The input power factor of 0.98 was calculated based on the division of the reactive kVA and the real kVA as indicated below which shows how well the converter is able to use the supplied power, without drawing much of re-triplex current from the grid. This is more so in the present complex power systems which require very high power factor in order to minimize losses and for stability of the system. A high power factor further also increases the utilization of existing infrastructure, more EVs can be charged at one time without stressing the system. They state that the total harmonic distortion of 5% is an indication that the converter generates less distorted waveform than the other competitors which is very important in shaping the electrical grid. Large values of THD cause heating of electrical components, deterioration of equipment, large losses and fines from the utilities. It also demonstrates that by implementing lower THD standards it enhances compatibility with the grid and creates the potential for the resonant LLC converter as an essential component of charging stations.

Experimental waveforms demonstrating Zero-Voltage Switching operation are presented to verify the soft-switching capability of the proposed LLC converter. The measured switch node voltage and gate drive signals confirm that the MOSFETs are turned on when the drain-to-source voltage approaches zero, thereby minimizing switching losses. Resonant tank current waveforms further validate proper resonant operation across the tested load range, supporting the high efficiency reported in the experimental results.

The switching frequency description has been revised for technical accuracy. In practical power electronic applications, LLC resonant converters typically operate in the range of 100 kHz to 500 kHz. Accordingly, the converter presented in this work operates within this frequency range, ensuring efficient power transfer while maintaining manageable switching losses. The previously stated value has been corrected to reflect realistic operating conditions.

Zero-Voltage Switching is a critical feature of the proposed LLC converter and is guaranteed over a wide operating range. ZVS is maintained for load conditions between 20% and 100% of rated load and across the full input voltage range of 180–260 V AC. Under light-load conditions below 20%, partial soft-switching is observed; however, the converter continues to operate with acceptable efficiency and stability. Experimental waveforms confirm that the MOSFET drain-to-source voltage falls to near zero prior to gate turn-on, validating ZVS operation at both nominal and worst-case input voltage conditions. This wide ZVS range significantly reduces switching losses and contributes to the measured peak efficiency of 95%.

### Comparison with traditional metrics

As seen from the results based on the above analysis, the proposed resonant LLC converter has significantly improved a number of performance metrics compared with the traditional diode bridge rectifiers. Generic systems are known to possess efficiencies below 90%, thus indicating high energy losses during charging. Moreover, in many cases the input power factors of these traditional systems are less than 0. 90 less efficient power utilization and high value of reactive power. These drawbacks are even more pronounced when the conventional chargers are utilized because they have high THD levels that can cause possible interruptions in grid reliability and increased operational expenses. On the other hand, the advanced resonant LLC converter shows not only superior traditional performance that exceeds the efficiency and power density of conventional rectifiers and converters, but also a more eco-friendly approach to charge EVs. The potentials for higher efficiency, unity power factor, and low total harmonic distortion situate this technology as the game-changer in today’s charging technology ecosystem. Interestingly, this research also suggests that use of resonant converters has a high probability of diffusion into the broader EV charging infrastructure that currently in the future in a way that will be beneficial for a greener economy. In order to make a fair comparison, the baseline system used in this paper is the one with a standard EV charger architecture of the diode bridge rectifier connected to non-isolated DC-DC converter without active power factor correction. These types of arrangements are typical of low cost charging systems and tend to have worse input current shaping and be more harboring of harmonic distortion. On the other hand, the suggested architecture uses a dynamic stage of PFC and an LLC resonant DC-DC converter, which allows to achieve better power quality and increase efficiency. The comparison hence draws attention on the advantage of substituting the traditional passive rectification phases by the current resonant converter-based networks. Table 2 shows that comparison between **Diode Bridge Rectifier and LLC Resonant Converter (Proposed)**.

**Table 2:** Performance comparison between conventional diode-rectifier charger and proposed PFC-LLC charger.

Parameter	Diode bridge rectifier	LLC resonant converter (proposed)
Efficiency (%)	65	95
Power Factor (PF)	0.78	0.98
THD (%)	22	4.7
Output Voltage Ripple (V)	12	2.5
Input Current Waveform	Distorted	Nearly sinusoidal
Switching Loss / ZVS	No ZVS	Verified ZVS

### Comparison with other converter topologies

Besides the comparison between the suggested converter and traditional systems of diode rectifier, there is also a need to compare the converters performance with other topologies of power converters commonly utilized in charging applications in EV. Converters classified as popular include flyback converters, phase-shifted full bridge (PSFB) converters and dual-active bridge (DAB) converters. Flyback converters are commonly applied to low power charging systems because they have a simple structure and are less costly but generally less efficient and most importantly the switching stress is harder to tolerate at higher power levels. Phase-shifted full bridge converters have higher efficiency and are typically applied in medium- to high-power operation but need more difficult control and more magnetic materials. Dual-active bridge converters allow the capability of bidirectional flow of power and high power density but with more complex circuits and more switching losses in special operating conditions. The LLC resonant converter has been suggested in this paper and handful of advantages associated to it are such as soft-switching characteristics, low switching losses, high efficiency, and high power densities. Table 3 gives a comparison of various converter topologies that are regularly used in EV charging systems.

**Table 3:** Comparison of different power converter topologies for EV charging applications.

Converter topology	Efficiency (%)	Switching loss	Power density	Control complexity	Typical application
Flyback Converter	80–88	High	Low	Simple	Low-power chargers
Phase Shifted Full Bridge (PSFB)	90–94	Moderate	Medium	Moderate	Medium-power chargers
Dual Active Bridge (DAB)	90–95	Moderate	High	Complex	Bidirectional chargers
Proposed LLC Resonant Converter	95	Low (ZVS)	High	Moderate	High-efficiency EV charging

As Table 3 indicates, the proposed LLC resonant converter represents a quintessential blend of high efficiency, soft-switching, and moderate complexity control, which is most appropriate in modern EV charging applications.

### Further development and implication of this line of research

Thus, several directions for further research are listed to advance the findings of this work further ahead. Another area that is still unexplored is refining the LLC converter, utilizing non-linear controls for optimization and employing passive and active techniques for adaptive rectification of the converter’s response under diverse load allude. Furthermore, the integration of renewable energy systems in charging the electric cars, such as solar or wind energy can expand the sustainability research area, and as the future work, consider the hybrid system charging by both conventional power grid and renewable energy recourses. Further, by applying machine learning approaches for the expansion of the model for predictive maintenance, more information about the kind of operations within the EV chargers might be ascertained. In this research, higher algorithms and volume of database may improve the effectiveness of predictive maintenance and reduce the duration of any downtime even more in the future. These strategies will help improve the charging infrastructure for EVs in the long run hence a boost in the employment of EVs hence a change in the transport sector from the conventional fuelled cars to the new fashioned EVs.

## Conclusions

Thus, this research manages to show how resonant LLC converter is indeed capable of improving PQ for EV chargers. The PFC front-end stage is used to make input power factor (0.98) nearly unified, whereas LLC converter is used to make isolated conversion between DC and DC. The Walters converters that have been discussed above achieve high levels of performance in terms of their input power factor of 0.98, total harmonic distortion of only 5%, and efficiency of 95%, and outperforming conventional diode bridge rectifiers greatly. The actual implementation used in experiments verified these characteristics and demonstrated superiority of the physical prototype in practice. In addition, a model of support vector regression for building the predictive maintenance with a 30% improvement in downtime for the chargers and reduce the performance problem. Besides, the results provided also demonstrate the benefits of the proposed converter design and further emphasize its importance to the establishment of solid efficient EV charging system which is finally going to spur the growth of the e-mobility and create fundamental basis for building more sustainable transportation system in the future.

## Data Availability

The datasets used and/or analysed during the current study available from the corresponding author on reasonable request.
